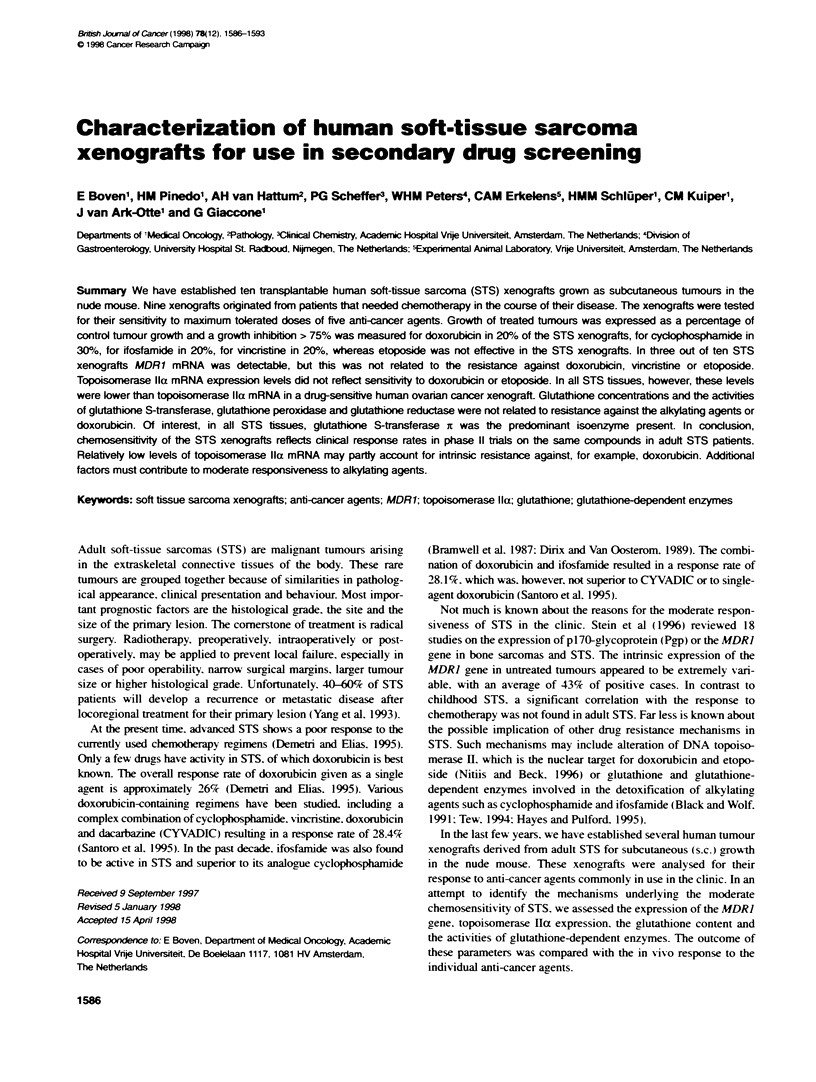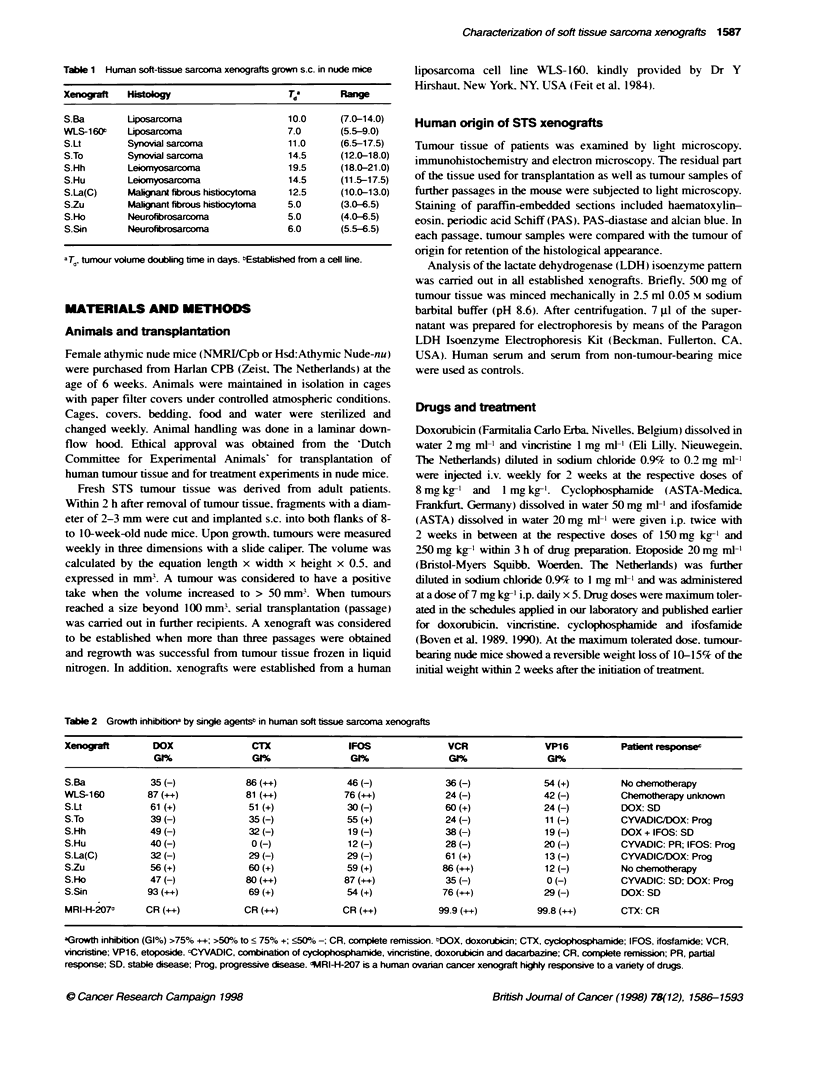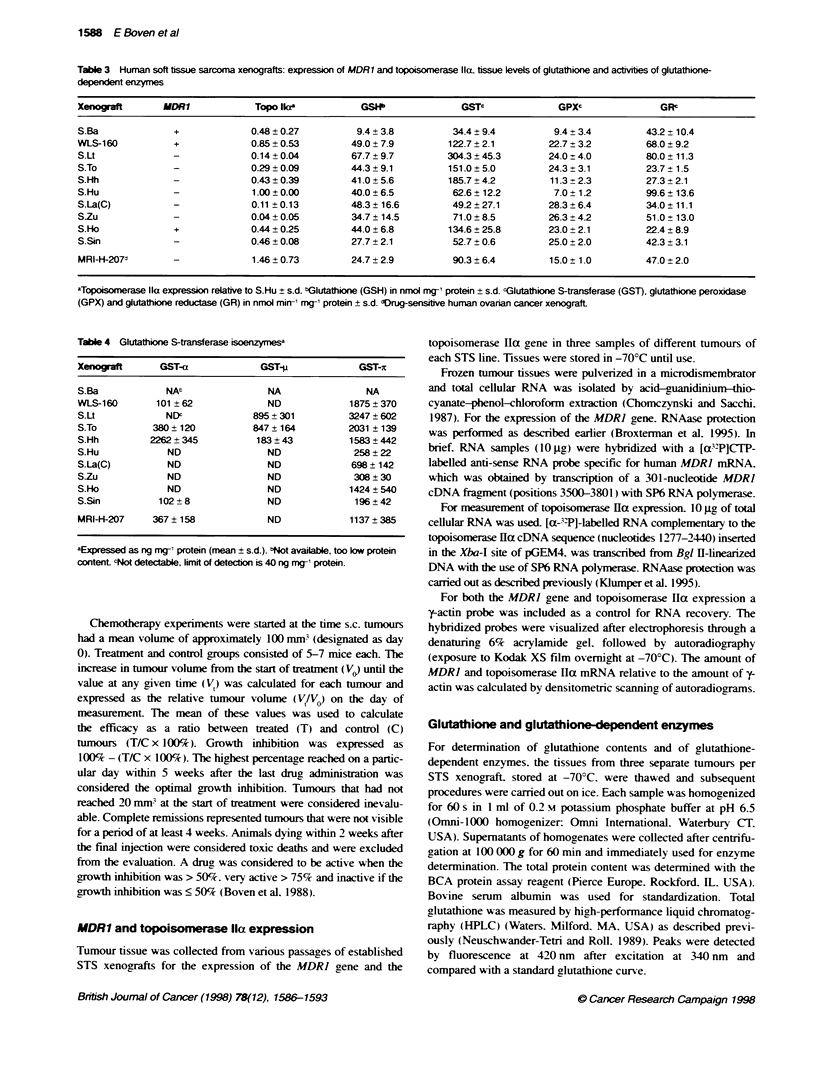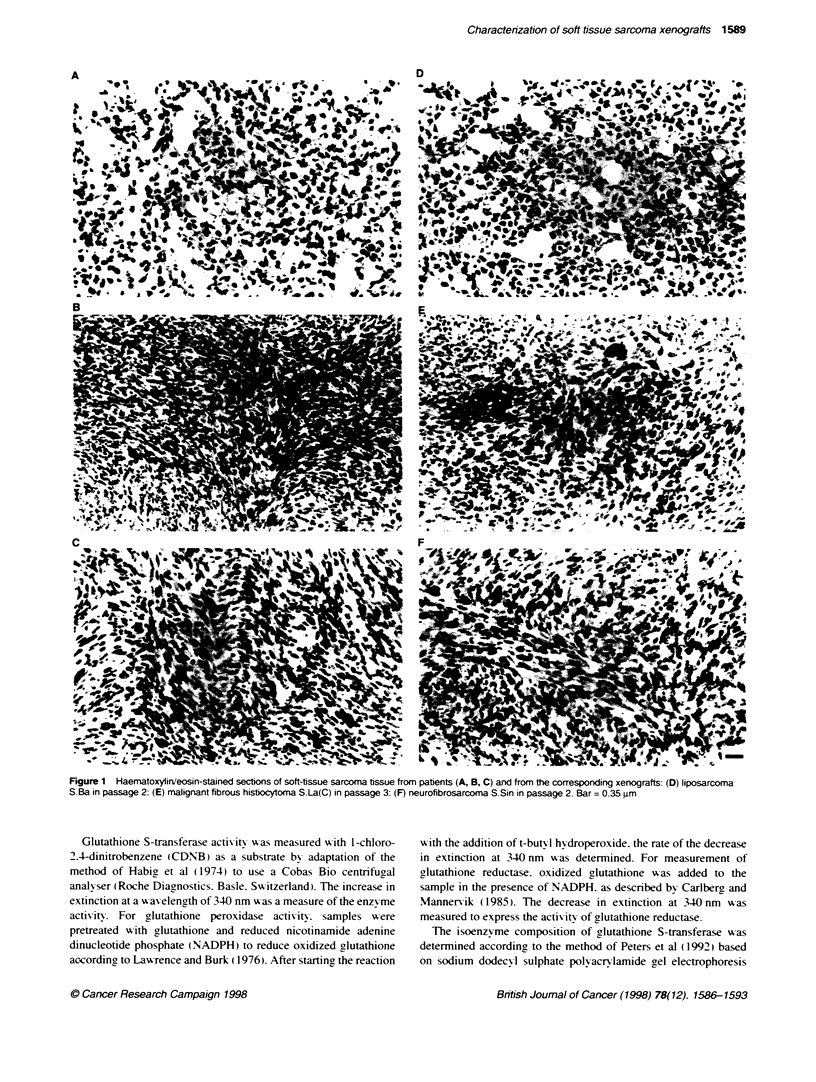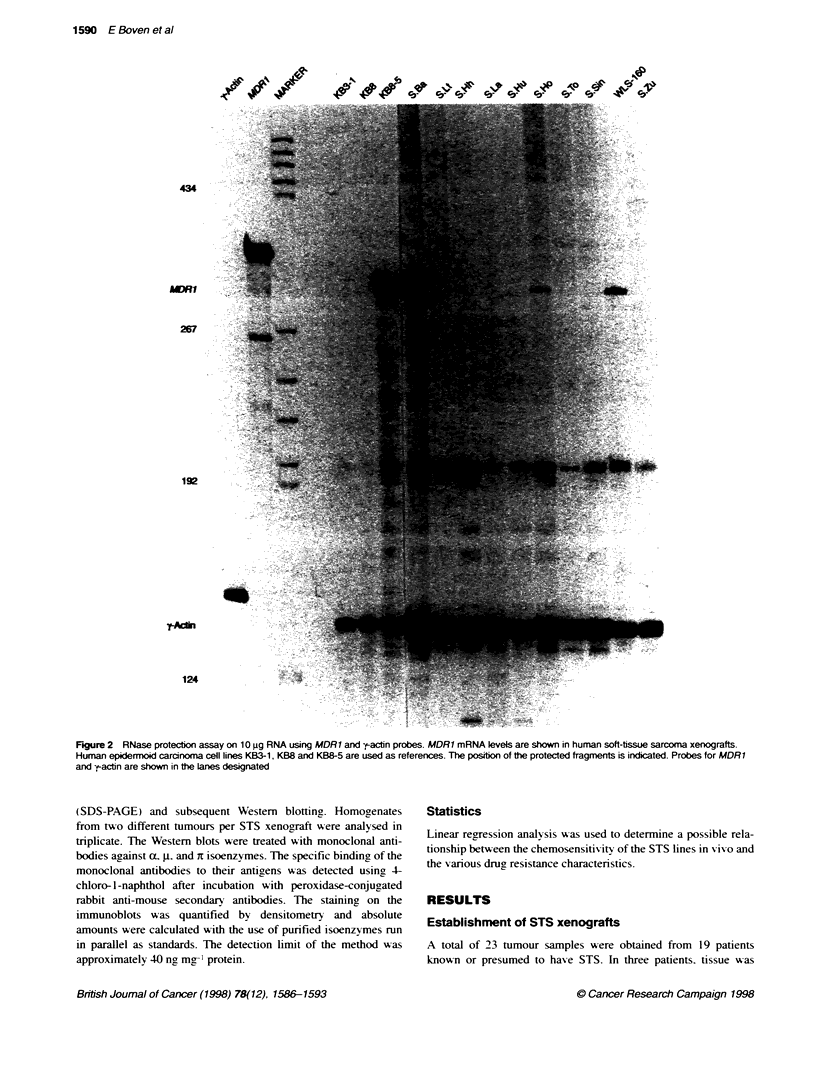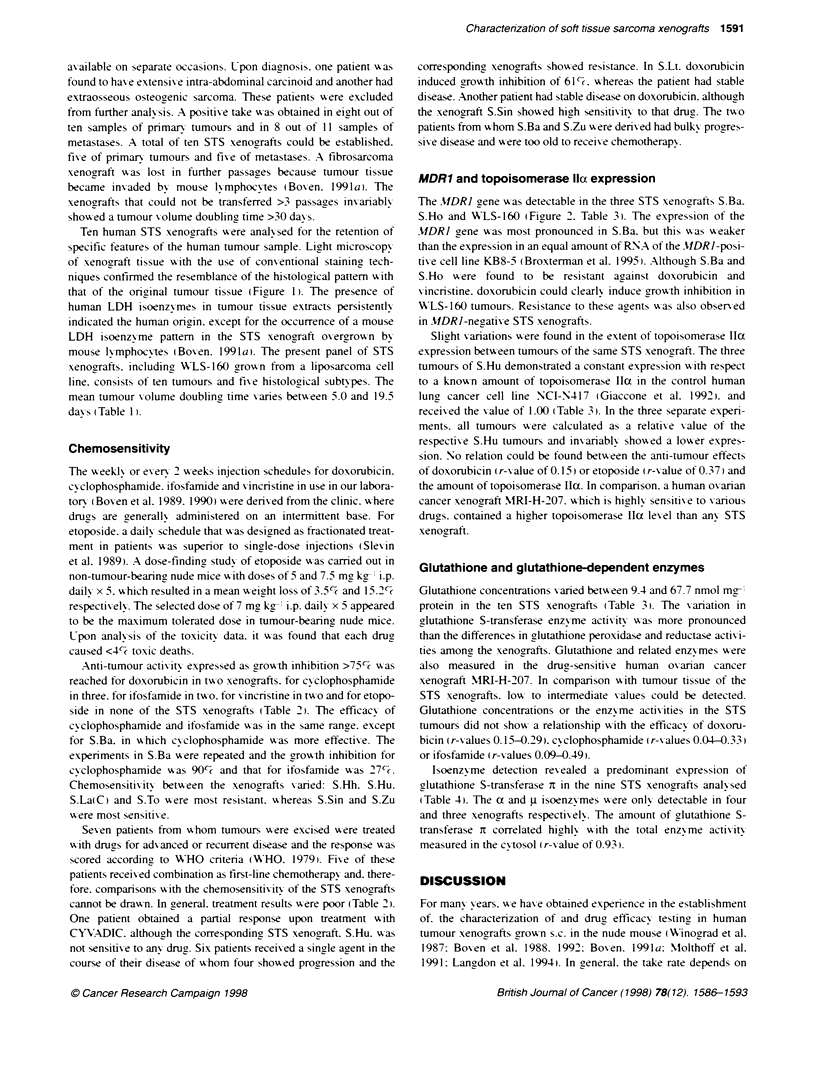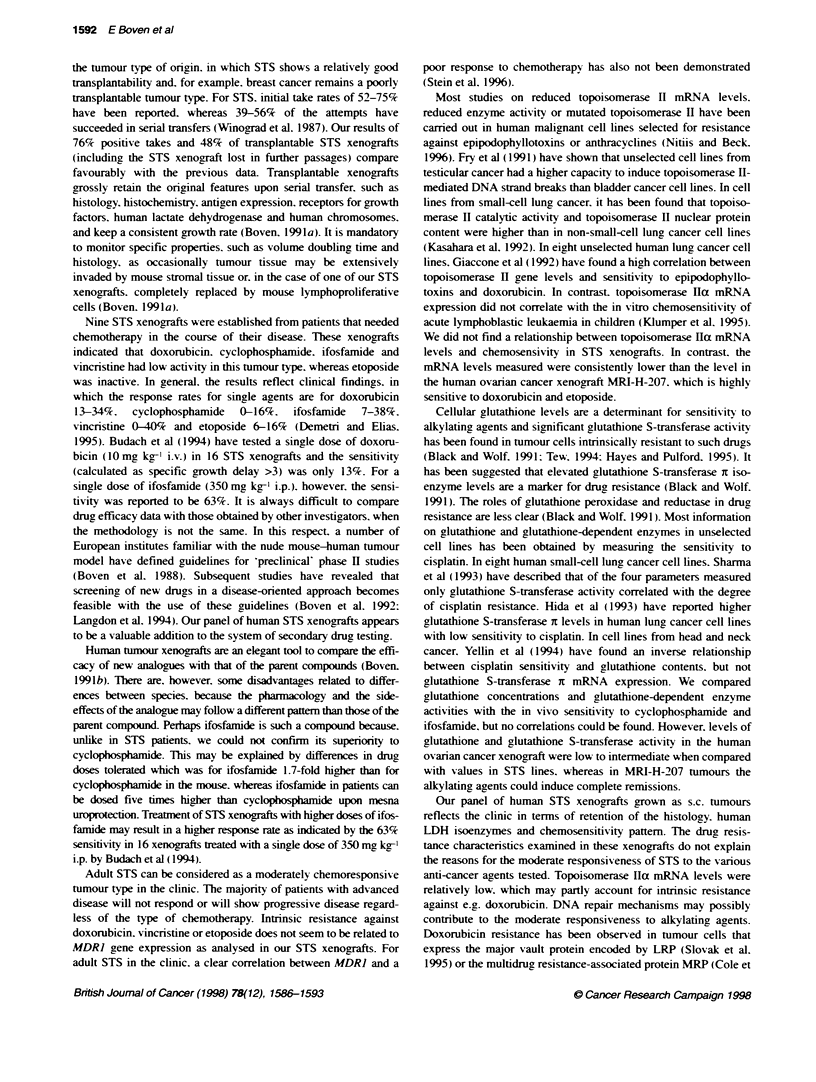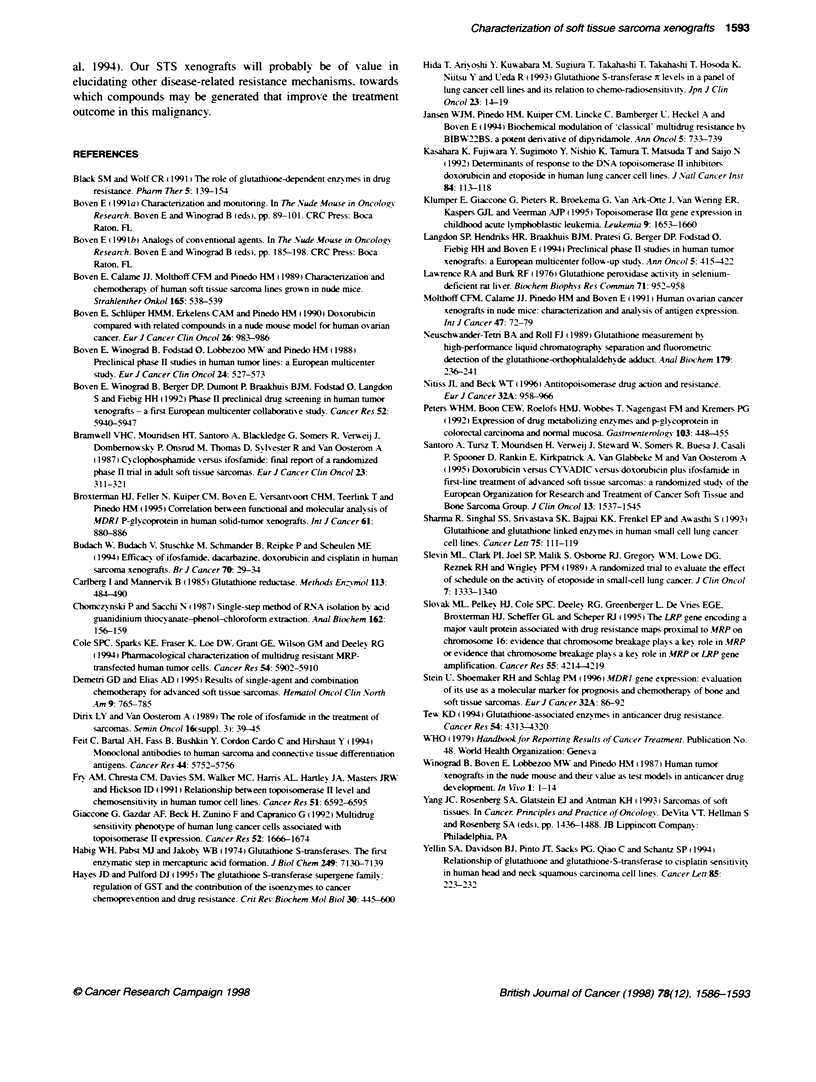# Characterization of human soft-tissue sarcoma xenografts for use in secondary drug screening.

**DOI:** 10.1038/bjc.1998.727

**Published:** 1998-12

**Authors:** E. Boven, H. M. Pinedo, A. H. van Hattum, P. G. Scheffer, W. H. Peters, C. A. Erkelens, H. M. Schlüper, C. M. Kuiper, J. van Ark-Otte, G. Giaccone

**Affiliations:** Department of Medical Oncology, Academic Hospital Vrije Universiteit, Amsterdam, The Netherlands.

## Abstract

**Images:**


British Journal of Cancer (1998) 78(12), 1586–1593
© 1998 Cancer Research Campaign

### Characterization of human soft-tissue sarcoma xenografts for use in secondary drug screening

**E Boven[1], HM Pinedo[1], AH van Hattum[2], PG Scheffer[3], WHM Peters[4], CAM Erkelens[5], HMM Schlüper[1], CM Kuiper[1], J van Ark-Otte[1] and G Giaccone[1]**

Departments of [1]Medical Oncology, [2]Pathology, [3]Clinical Chemistry, Academic Hospital Vrije Universiteit, Amsterdam, The Netherlands; [4]Division of Gastroenterology, University Hospital St. Radboud, Nijmegen, The Netherlands; [5]Experimental Animal Laboratory, Vrije Universiteit, Amsterdam, The Netherlands

**Summary** We have established ten transplantable human soft-tissue sarcoma (STS) xenografts grown as subcutaneous tumours in the nude mouse. Nine xenografts originated from patients that needed chemotherapy in the course of their disease. The xenografts were tested for their sensitivity to maximum tolerated doses of five anti-cancer agents. Growth of treated tumours was expressed as a percentage of control tumour growth and a growth inhibition > 75% was measured for doxorubicin in 20% of the STS xenografts, for cyclophosphamide in 30%, for ifosfamide in 20%, for vincristine in 20%, whereas etoposide was not effective in the STS xenografts. In three out of ten STS xenografts *MDR1* mRNA was detectable, but this was not related to the resistance against doxorubicin, vincristine or etoposide. Topoisomerase IIα mRNA expression levels did not reflect sensitivity to doxorubicin or etoposide. In all STS tissues, however, these levels were lower than topoisomerase IIα mRNA in a drug-sensitive human ovarian cancer xenograft. Glutathione concentrations and the activities of glutathione S-transferase, glutathione peroxidase and glutathione reductase were not related to resistance against the alkylating agents or doxorubicin. Of interest, in all STS tissues, glutathione S-transferase π was the predominant isoenzyme present. In conclusion, chemosensitivity of the STS xenografts reflects clinical response rates in phase II trials on the same compounds in adult STS patients. Relatively low levels of topoisomerase IIα mRNA may partly account for intrinsic resistance against, for example, doxorubicin. Additional factors must contribute to moderate responsiveness to alkylating agents.

**Keywords:** soft tissue sarcoma xenografts; anti-cancer agents; *MDR1*; topoisomerase IIα; glutathione; glutathione-dependent enzymes

Adult soft-tissue sarcomas (STS) are malignant tumours arising in the extraskeletal connective tissues of the body. These rare tumours are grouped together because of similarities in pathological appearance, clinical presentation and behaviour. Most important prognostic factors are the histological grade, the site and the size of the primary lesion. The cornerstone of treatment is radical surgery. Radiotherapy, preoperatively, intraoperatively or postoperatively, may be applied to prevent local failure, especially in cases of poor operability, narrow surgical margins, larger tumour size or higher histological grade. Unfortunately, 40–60% of STS patients will develop a recurrence or metastatic disease after locoregional treatment for their primary lesion (Yang et al, 1993).

At the present time, advanced STS shows a poor response to the currently used chemotherapy regimens (Demetri and Elias, 1995). Only a few drugs have activity in STS, of which doxorubicin is best known. The overall response rate of doxorubicin given as a single agent is approximately 26% (Demetri and Elias, 1995). Various doxorubicin-containing regimens have been studied, including a complex combination of cyclophosphamide, vincristine, doxorubicin and dacarbazine (CYVADIC) resulting in a response rate of 28.4% (Santoro et al, 1995). In the past decade, ifosfamide was also found to be active in STS and superior to its analogue cyclophosphamide (Bramwell et al, 1987; Dirix and Van Oosterom, 1989). The combination of doxorubicin and ifosfamide resulted in a response rate of 28.1%, which was, however, not superior to CYVADIC or to single-agent doxorubicin (Santoro et al, 1995).

Not much is known about the reasons for the moderate responsiveness of STS in the clinic. Stein et al (1996) reviewed 18 studies on the expression of p170-glycoprotein (Pgp) or the *MDR1* gene in bone sarcomas and STS. The intrinsic expression of the *MDR1* gene in untreated tumours appeared to be extremely variable, with an average of 43% of positive cases. In contrast to childhood STS, a significant correlation with the response to chemotherapy was not found in adult STS. Far less is known about the possible implication of other drug resistance mechanisms in STS. Such mechanisms may include alteration of DNA topoisomerase II, which is the nuclear target for doxorubicin and etoposide (Nitiis and Beck, 1996) or glutathione and glutathione-dependent enzymes involved in the detoxification of alkylating agents such as cyclophosphamide and ifosfamide (Black and Wolf, 1991; Tew, 1994; Hayes and Pulford, 1995).

In the last few years, we have established several human tumour xenografts derived from adult STS for subcutaneous (s.c.) growth in the nude mouse. These xenografts were analysed for their response to anti-cancer agents commonly in use in the clinic. In an attempt to identify the mechanisms underlying the moderate chemosensitivity of STS, we assessed the expression of the *MDR1* gene, topoisomerase IIα expression, the glutathione content and the activities of glutathione-dependent enzymes. The outcome of these parameters was compared with the in vivo response to the individual anti-cancer agents.

*Received 9 September 1997*
*Revised 5 January 1998*
*Accepted 15 April 1998*

*Correspondence to:* E Boven, Department of Medical Oncology, Academic Hospital Vrije Universiteit, De Boelelaan 1117, 1081 HV Amsterdam, The Netherlands

**Table 1** Human soft-tissue sarcoma xenografts grown s.c. in nude mice

| Xenograft | Histology | $T_d$[a] | Range |
|---|---|---|---|
| S.Ba | Liposarcoma | 10.0 | (7.0–14.0) |
| WLS-160[b] | Liposarcoma | 7.0 | (5.5–9.0) |
| S.Lt | Synovial sarcoma | 11.0 | (6.5–17.5) |
| S.To | Synovial sarcoma | 14.5 | (12.0–18.0) |
| S.Hh | Leiomyosarcoma | 19.5 | (18.0–21.0) |
| S.Hu | Leiomyosarcoma | 14.5 | (11.5–17.5) |
| S.La(C) | Malignant fibrous histiocytoma | 12.5 | (10.0–13.0) |
| S.Zu | Malignant fibrous histiocytoma | 5.0 | (3.0–6.5) |
| S.Ho | Neurofibrosarcoma | 5.0 | (4.0–6.5) |
| S.Sin | Neurofibrosarcoma | 6.0 | (5.5–6.5) |

[a]$T_d$, tumour volume doubling time in days. [b]Established from a cell line.

### MATERIALS AND METHODS

### Animals and transplantation

Female athymic nude mice (NMRI/Cpb or Hsd:Athymic Nude-*nu*) were purchased from Harlan CPB (Zeist, The Netherlands) at the age of 6 weeks. Animals were maintained in isolation in cages with paper filter covers under controlled atmospheric conditions. Cages, covers, bedding, food and water were sterilized and changed weekly. Animal handling was done in a laminar down-flow hood. Ethical approval was obtained from the 'Dutch Committee for Experimental Animals' for transplantation of human tumour tissue and for treatment experiments in nude mice.

Fresh STS tumour tissue was derived from adult patients. Within 2 h after removal of tumour tissue, fragments with a diameter of 2–3 mm were cut and implanted s.c. into both flanks of 8- to 10-week-old nude mice. Upon growth, tumours were measured weekly in three dimensions with a slide caliper. The volume was calculated by the equation length × width × height × 0.5, and expressed in $mm^3$. A tumour was considered to have a positive take when the volume increased to > 50 $mm^3$. When tumours reached a size beyond 100 $mm^3$, serial transplantation (passage) was carried out in further recipients. A xenograft was considered to be established when more than three passages were obtained and regrowth was successful from tumour tissue frozen in liquid nitrogen. In addition, xenografts were established from a human liposarcoma cell line WLS-160, kindly provided by Dr Y Hirshaut, New York, NY, USA (Feit et al, 1984).

### Human origin of STS xenografts

Tumour tissue of patients was examined by light microscopy, immunohistochemistry and electron microscopy. The residual part of the tissue used for transplantation as well as tumour samples of further passages in the mouse were subjected to light microscopy. Staining of paraffin-embedded sections included haematoxylin–eosin, periodic acid Schiff (PAS), PAS-diastase and alcian blue. In each passage, tumour samples were compared with the tumour of origin for retention of the histological appearance.

Analysis of the lactate dehydrogenase (LDH) isoenzyme pattern was carried out in all established xenografts. Briefly, 500 mg of tumour tissue was minced mechanically in 2.5 ml 0.05 M sodium barbital buffer (pH 8.6). After centrifugation, 7 µl of the supernatant was prepared for electrophoresis by means of the Paragon LDH Isoenzyme Electrophoresis Kit (Beckman, Fullerton, CA, USA). Human serum and serum from non-tumour-bearing mice were used as controls.

### Drugs and treatment

Doxorubicin (Farmitalia Carlo Erba, Nivelles, Belgium) dissolved in water 2 mg $ml^{-1}$ and vincristine 1 mg $ml^{-1}$ (Eli Lilly, Nieuwegein, The Netherlands) diluted in sodium chloride 0.9% to 0.2 mg $ml^{-1}$ were injected i.v. weekly for 2 weeks at the respective doses of 8 mg $kg^{-1}$ and 1 mg $kg^{-1}$. Cyclophosphamide (ASTA-Medica, Frankfurt, Germany) dissolved in water 50 mg $ml^{-1}$ and ifosfamide (ASTA) dissolved in water 20 mg $ml^{-1}$ were given i.p. twice with 2 weeks in between at the respective doses of 150 mg $kg^{-1}$ and 250 mg $kg^{-1}$ within 3 h of drug preparation. Etoposide 20 mg $ml^{-1}$ (Bristol-Myers Squibb, Woerden, The Netherlands) was further diluted in sodium chloride 0.9% to 1 mg $ml^{-1}$ and was administered at a dose of 7 mg $kg^{-1}$ i.p. daily × 5. Drug doses were maximum tolerated in the schedules applied in our laboratory and published earlier for doxorubicin, vincristine, cyclophosphamide and ifosfamide (Boven et al, 1989, 1990). At the maximum tolerated dose, tumour-bearing nude mice showed a reversible weight loss of 10–15% of the initial weight within 2 weeks after the initiation of treatment.

**Table 2** Growth inhibition[a] by single agents[b] in human soft tissue sarcoma xenografts

| Xenograft | DOX GI% | CTX GI% | IFOS GI% | VCR GI% | VP16 GI% | Patient response[c] |
|---|---|---|---|---|---|---|
| S.Ba | 35 (–) | 86 (++) | 46 (–) | 36 (–) | 54 (+) | No chemotherapy |
| WLS-160 | 87 (++) | 81 (++) | 76 (++) | 24 (–) | 42 (–) | Chemotherapy unknown |
| S.Lt | 61 (+) | 51 (+) | 30 (–) | 60 (+) | 24 (–) | DOX: SD |
| S.To | 39 (–) | 35 (–) | 55 (+) | 24 (–) | 11 (–) | CYVADIC/DOX: Prog |
| S.Hh | 49 (–) | 32 (–) | 19 (–) | 38 (–) | 19 (–) | DOX + IFOS: SD |
| S.Hu | 40 (–) | 0 (–) | 12 (–) | 28 (–) | 20 (–) | CYVADIC: PR; IFOS: Prog |
| S.La(C) | 32 (–) | 29 (–) | 29 (–) | 61 (+) | 13 (–) | CYVADIC/DOX: Prog |
| S.Zu | 56 (+) | 60 (+) | 59 (+) | 86 (++) | 12 (–) | No chemotherapy |
| S.Ho | 47 (–) | 80 (++) | 87 (++) | 35 (–) | 0 (–) | CYVADIC: SD; DOX: Prog |
| S.Sin | 93 (++) | 69 (+) | 54 (+) | 76 (++) | 29 (–) | DOX: SD |
| MRI-H-207[d] | CR (++) | CR (++) | CR (++) | 99.9 (++) | 99.8 (++) | CTX: CR |

[a]Growth inhibition (GI%) >75% ++; >50% to ≤ 75% +; ≤50% –; CR, complete remission. [b]DOX, doxorubicin; CTX, cyclophosphamide; IFOS, ifosfamide; VCR, vincristine; VP16, etoposide. [c]CYVADIC, combination of cyclophosphamide, vincristine, doxorubicin and dacarbazine; CR, complete remission; PR, partial response; SD, stable disease; Prog, progressive disease. [d]MRI-H-207 is a human ovarian cancer xenograft highly responsive to a variety of drugs.

© Cancer Research Campaign 1998 

**Table 3** Human soft tissue sarcoma xenografts: expression of *MDR1* and topoisomerase IIα, tissue levels of glutathione and activities of glutathione-dependent enzymes

| Xenograft | *MDR1* | Topo IIα[a] | GSH[b] | GST[c] | GPX[c] | GR[c] |
|---|---|---|---|---|---|---|
| S.Ba | + | 0.48 ± 0.27 | 9.4 ± 3.8 | 34.4 ± 9.4 | 9.4 ± 3.4 | 43.2 ± 10.4 |
| WLS-160 | + | 0.85 ± 0.53 | 49.0 ± 7.9 | 122.7 ± 2.1 | 22.7 ± 3.2 | 68.0 ± 9.2 |
| S.Lt | – | 0.14 ± 0.04 | 67.7 ± 9.7 | 304.3 ± 45.3 | 24.0 ± 4.0 | 80.0 ± 11.3 |
| S.To | – | 0.29 ± 0.09 | 44.3 ± 9.1 | 151.0 ± 5.0 | 24.3 ± 3.1 | 23.7 ± 1.5 |
| S.Hh | – | 0.43 ± 0.39 | 41.0 ± 5.6 | 185.7 ± 4.2 | 11.3 ± 2.3 | 27.3 ± 2.1 |
| S.Hu | – | 1.00 ± 0.00 | 40.0 ± 6.5 | 62.6 ± 12.2 | 7.0 ± 1.2 | 99.6 ± 13.6 |
| S.La(C) | – | 0.11 ± 0.13 | 48.3 ± 16.6 | 49.2 ± 27.1 | 28.3 ± 6.4 | 34.0 ± 11.1 |
| S.Zu | – | 0.04 ± 0.05 | 34.7 ± 14.5 | 71.0 ± 8.5 | 26.3 ± 4.2 | 51.0 ± 13.0 |
| S.Ho | + | 0.44 ± 0.25 | 44.0 ± 6.8 | 134.6 ± 25.8 | 23.0 ± 2.1 | 22.4 ± 8.9 |
| S.Sin | – | 0.46 ± 0.08 | 27.7 ± 2.1 | 52.7 ± 0.6 | 25.0 ± 2.0 | 42.3 ± 3.1 |
| MRI-H-207[d] | – | 1.46 ± 0.73 | 24.7 ± 2.9 | 90.3 ± 6.4 | 15.0 ± 1.0 | 47.0 ± 2.0 |

[a]Topoisomerase IIα expression relative to S.Hu ± s.d. [b]Glutathione (GSH) in nmol mg$^{-1}$ protein ± s.d. [c]Glutathione S-transferase (GST), glutathione peroxidase (GPX) and glutathione reductase (GR) in nmol min$^{-1}$ mg$^{-1}$ protein ± s.d. [d]Drug-sensitive human ovarian cancer xenograft.

**Table 4** Glutathione S-transferase isoenzymes[a]

| Xenograft | GST-α | GST-μ | GST-π |
|---|---|---|---|
| S.Ba | NA[b] | NA | NA |
| WLS-160 | 101 ± 62 | ND | 1875 ± 370 |
| S.Lt | ND[c] | 895 ± 301 | 3247 ± 602 |
| S.To | 380 ± 120 | 847 ± 164 | 2031 ± 139 |
| S.Hh | 2262 ± 345 | 183 ± 43 | 1583 ± 442 |
| S.Hu | ND | ND | 258 ± 22 |
| S.La(C) | ND | ND | 698 ± 142 |
| S.Zu | ND | ND | 308 ± 30 |
| S.Ho | ND | ND | 1424 ± 540 |
| S.Sin | 102 ± 8 | ND | 196 ± 42 |
| MRI-H-207 | 367 ± 158 | ND | 1137 ± 385 |

[a]Expressed as ng mg$^{-1}$ protein (mean ± s.d.). [b]Not available, too low protein content. [c]Not detectable, limit of detection is 40 ng mg$^{-1}$ protein.

Chemotherapy experiments were started at the time s.c. tumours had a mean volume of approximately 100 mm$^3$ (designated as day 0). Treatment and control groups consisted of 5–7 mice each. The increase in tumour volume from the start of treatment ($V_0$) until the value at any given time ($V_t$) was calculated for each tumour and expressed as the relative tumour volume ($V_t/V_0$) on the day of measurement. The mean of these values was used to calculate the efficacy as a ratio between treated (T) and control (C) tumours (T/C × 100%). Growth inhibition was expressed as 100% – (T/C × 100%). The highest percentage reached on a particular day within 5 weeks after the last drug administration was considered the optimal growth inhibition. Tumours that had not reached 20 mm$^3$ at the start of treatment were considered inevaluable. Complete remissions represented tumours that were not visible for a period of at least 4 weeks. Animals dying within 2 weeks after the final injection were considered toxic deaths and were excluded from the evaluation. A drug was considered to be active when the growth inhibition was > 50%, very active > 75% and inactive if the growth inhibition was ≤ 50% (Boven et al, 1988).

### *MDR1* and topoisomerase IIα expression

Tumour tissue was collected from various passages of established STS xenografts for the expression of the *MDR1* gene and the topoisomerase IIα gene in three samples of different tumours of each STS line. Tissues were stored in –70°C until use.

Frozen tumour tissues were pulverized in a microdismembrator and total cellular RNA was isolated by acid–guanidinium–thiocyanate–phenol–chloroform extraction (Chomczynski and Sacchi, 1987). For the expression of the *MDR1* gene, RNAase protection was performed as described earlier (Broxterman et al, 1995). In brief, RNA samples (10 μg) were hybridized with a [α-$^{32}$P]CTP-labelled anti-sense RNA probe specific for human *MDR1* mRNA, which was obtained by transcription of a 301-nucleotide *MDR1* cDNA fragment (positions 3500–3801) with SP6 RNA polymerase.

For measurement of topoisomerase IIα expression, 10 μg of total cellular RNA was used. [α-$^{32}$P]-labelled RNA complementary to the topoisomerase IIα cDNA sequence (nucleotides 1277–2440) inserted in the *Xba*-I site of pGEM4, was transcribed from *Bgl* II-linearized DNA with the use of SP6 RNA polymerase. RNAase protection was carried out as described previously (Klumper et al, 1995).

For both the *MDR1* gene and topoisomerase IIα expression a γ-actin probe was included as a control for RNA recovery. The hybridized probes were visualized after electrophoresis through a denaturing 6% acrylamide gel, followed by autoradiography (exposure to Kodak XS film overnight at –70°C). The amount of *MDR1* and topoisomerase IIα mRNA relative to the amount of γ-actin was calculated by densitometric scanning of autoradiograms.

### Glutathione and glutathione-dependent enzymes

For determination of glutathione contents and of glutathione-dependent enzymes, the tissues from three separate tumours per STS xenograft, stored at –70°C, were thawed and subsequent procedures were carried out on ice. Each sample was homogenized for 60 s in 1 ml of 0.2 M potassium phosphate buffer at pH 6.5 (Omni-1000 homogenizer; Omni International, Waterbury CT, USA). Supernatants of homogenates were collected after centrifugation at 100 000 *g* for 60 min and immediately used for enzyme determination. The total protein content was determined with the BCA protein assay reagent (Pierce Europe, Rockford, IL, USA). Bovine serum albumin was used for standardization. Total glutathione was measured by high-performance liquid chromatography (HPLC) (Waters, Milford, MA, USA) as described previously (Neuschwander-Tetri and Roll, 1989). Peaks were detected by fluorescence at 420 nm after excitation at 340 nm and compared with a standard glutathione curve.

© Cancer Research Campaign 1998

**Figure 1** Haematoxylin/eosin-stained sections of soft-tissue sarcoma tissue from patients (**A, B, C**) and from the corresponding xenografts: (**D**) liposarcoma S.Ba in passage 2: (**E**) malignant fibrous histiocytoma S.La(C) in passage 3: (**F**) neurofibrosarcoma S.Sin in passage 2. Bar = 0.35 μm

Glutathione S-transferase activity was measured with 1-chloro-2,4-dinitrobenzene (CDNB) as a substrate by adaptation of the method of Habig et al (1974) to use a Cobas Bio centrifugal analyser (Roche Diagnostics, Basle, Switzerland). The increase in extinction at a wavelength of 340 nm was a measure of the enzyme activity. For glutathione peroxidase activity, samples were pretreated with glutathione and reduced nicotinamide adenine dinucleotide phosphate (NADPH) to reduce oxidized glutathione according to Lawrence and Burk (1976). After starting the reaction with the addition of t-butyl hydroperoxide, the rate of the decrease in extinction at 340 nm was determined. For measurement of glutathione reductase, oxidized glutathione was added to the sample in the presence of NADPH, as described by Carlberg and Mannervik (1985). The decrease in extinction at 340 nm was measured to express the activity of glutathione reductase.

The isoenzyme composition of glutathione S-transferase was determined according to the method of Peters et al (1992) based on sodium dodecyl sulphate polyacrylamide gel electrophoresis

© Cancer Research Campaign 1998

**Figure 2** RNase protection assay on 10 μg RNA using *MDR1* and γ-actin probes. *MDR1* mRNA levels are shown in human soft-tissue sarcoma xenografts. Human epidermoid carcinoma cell lines KB3-1, KB8 and KB8-5 are used as references. The position of the protected fragments is indicated. Probes for *MDR1* and γ-actin are shown in the lanes designated

(SDS-PAGE) and subsequent Western blotting. Homogenates from two different tumours per STS xenograft were analysed in triplicate. The Western blots were treated with monoclonal antibodies against α, μ, and π isoenzymes. The specific binding of the monoclonal antibodies to their antigens was detected using 4-chloro-1-naphthol after incubation with peroxidase-conjugated rabbit anti-mouse secondary antibodies. The staining on the immunoblots was quantified by densitometry and absolute amounts were calculated with the use of purified isoenzymes run in parallel as standards. The detection limit of the method was approximately 40 ng $mg^{-1}$ protein.

### Statistics

Linear regression analysis was used to determine a possible relationship between the chemosensitivity of the STS lines in vivo and the various drug resistance characteristics.

### RESULTS

### Establishment of STS xenografts

A total of 23 tumour samples were obtained from 19 patients known or presumed to have STS. In three patients, tissue was

© Cancer Research Campaign 1998

available on separate occasions. Upon diagnosis, one patient was found to have extensive intra-abdominal carcinoid and another had extraosseous osteogenic sarcoma. These patients were excluded from further analysis. A positive take was obtained in eight out of ten samples of primary tumours and in 8 out of 11 samples of metastases. A total of ten STS xenografts could be established, five of primary tumours and five of metastases. A fibrosarcoma xenograft was lost in further passages because tumour tissue became invaded by mouse lymphocytes (Boven, 1991*a*). The xenografts that could not be transferred >3 passages invariably showed a tumour volume doubling time >30 days.

Ten human STS xenografts were analysed for the retention of specific features of the human tumour sample. Light microscopy of xenograft tissue with the use of conventional staining techniques confirmed the resemblance of the histological pattern with that of the original tumour tissue (Figure 1). The presence of human LDH isoenzymes in tumour tissue extracts persistently indicated the human origin, except for the occurrence of a mouse LDH isoenzyme pattern in the STS xenograft overgrown by mouse lymphocytes (Boven, 1991*a*). The present panel of STS xenografts, including WLS-160 grown from a liposarcoma cell line, consists of ten tumours and five histological subtypes. The mean tumour volume doubling time varies between 5.0 and 19.5 days (Table 1).

### Chemosensitivity

The weekly or every 2 weeks injection schedules for doxorubicin, cyclophosphamide, ifosfamide and vincristine in use in our laboratory (Boven et al, 1989, 1990) were derived from the clinic, where drugs are generally administered on an intermittent base. For etoposide, a daily schedule that was designed as fractionated treatment in patients was superior to single-dose injections (Slevin et al, 1989). A dose-finding study of etoposide was carried out in non-tumour-bearing nude mice with doses of 5 and 7.5 mg $kg^{-1}$ i.p. daily × 5, which resulted in a mean weight loss of 3.5% and 15.2% respectively. The selected dose of 7 mg $kg^{-1}$ i.p. daily × 5 appeared to be the maximum tolerated dose in tumour-bearing nude mice. Upon analysis of the toxicity data, it was found that each drug caused <4% toxic deaths.

Anti-tumour activity expressed as growth inhibition >75% was reached for doxorubicin in two xenografts, for cyclophosphamide in three, for ifosfamide in two, for vincristine in two and for etoposide in none of the STS xenografts (Table 2). The efficacy of cyclophosphamide and ifosfamide was in the same range, except for S.Ba, in which cyclophosphamide was more effective. The experiments in S.Ba were repeated and the growth inhibition for cyclophosphamide was 90% and that for ifosfamide was 27%. Chemosensitivity between the xenografts varied: S.Hh, S.Hu, S.La(C) and S.To were most resistant, whereas S.Sin and S.Zu were most sensitive.

Seven patients from whom tumours were excised were treated with drugs for advanced or recurrent disease and the response was scored according to WHO criteria (WHO, 1979). Five of these patients received combination as first-line chemotherapy and, therefore, comparisons with the chemosensitivity of the STS xenografts cannot be drawn. In general, treatment results were poor (Table 2). One patient obtained a partial response upon treatment with CYVADIC, although the corresponding STS xenograft, S.Hu, was not sensitive to any drug. Six patients received a single agent in the course of their disease of whom four showed progression and the corresponding xenografts showed resistance. In S.Lt, doxorubicin induced growth inhibition of 61%, whereas the patient had stable disease. Another patient had stable disease on doxorubicin, although the xenograft S.Sin showed high sensitivity to that drug. The two patients from whom S.Ba and S.Zu were derived had bulky progressive disease and were too old to receive chemotherapy.

### *MDR1* and topoisomerase IIα expression

The *MDR1* gene was detectable in the three STS xenografts S.Ba, S.Ho and WLS-160 (Figure 2, Table 3). The expression of the *MDR1* gene was most pronounced in S.Ba, but this was weaker than the expression in an equal amount of RNA of the *MDR1*-positive cell line KB8-5 (Broxterman et al, 1995). Although S.Ba and S.Ho were found to be resistant against doxorubicin and vincristine, doxorubicin could clearly induce growth inhibition in WLS-160 tumours. Resistance to these agents was also observed in *MDR1*-negative STS xenografts.

Slight variations were found in the extent of topoisomerase IIα expression between tumours of the same STS xenograft. The three tumours of S.Hu demonstrated a constant expression with respect to a known amount of topoisomerase IIα in the control human lung cancer cell line NCI-N417 (Giaccone et al, 1992), and received the value of 1.00 (Table 3). In the three separate experiments, all tumours were calculated as a relative value of the respective S.Hu tumours and invariably showed a lower expression. No relation could be found between the anti-tumour effects of doxorubicin (*r*-value of 0.15) or etoposide (*r*-value of 0.37) and the amount of topoisomerase IIα. In comparison, a human ovarian cancer xenograft MRI-H-207, which is highly sensitive to various drugs, contained a higher topoisomerase IIα level than any STS xenograft.

### Glutathione and glutathione-dependent enzymes

Glutathione concentrations varied between 9.4 and 67.7 nmol $mg^{-1}$ protein in the ten STS xenografts (Table 3). The variation in glutathione S-transferase enzyme activity was more pronounced than the differences in glutathione peroxidase and reductase activities among the xenografts. Glutathione and related enzymes were also measured in the drug-sensitive human ovarian cancer xenograft MRI-H-207. In comparison with tumour tissue of the STS xenografts, low to intermediate values could be detected. Glutathione concentrations or the enzyme activities in the STS tumours did not show a relationship with the efficacy of doxorubicin (*r*-values 0.15–0.29), cyclophosphamide (*r*-values 0.04–0.33) or ifosfamide (*r*-values 0.09–0.49).

Isoenzyme detection revealed a predominant expression of glutathione S-transferase π in the nine STS xenografts analysed (Table 4). The α and μ isoenzymes were only detectable in four and three xenografts respectively. The amount of glutathione S-transferase π correlated highly with the total enzyme activity measured in the cytosol (*r*-value of 0.93).

### DISCUSSION

For many years, we have obtained experience in the establishment of, the characterization of and drug efficacy testing in human tumour xenografts grown s.c. in the nude mouse (Winograd et al, 1987; Boven et al, 1988, 1992; Boven, 1991*a*; Molthoff et al, 1991; Langdon et al, 1994). In general, the take rate depends on

© Cancer Research Campaign 1998

the tumour type of origin, in which STS shows a relatively good transplantability and, for example, breast cancer remains a poorly transplantable tumour type. For STS, initial take rates of 52–75% have been reported, whereas 39–56% of the attempts have succeeded in serial transfers (Winograd et al, 1987). Our results of 76% positive takes and 48% of transplantable STS xenografts (including the STS xenograft lost in further passages) compare favourably with the previous data. Transplantable xenografts grossly retain the original features upon serial transfer, such as histology, histochemistry, antigen expression, receptors for growth factors, human lactate dehydrogenase and human chromosomes, and keep a consistent growth rate (Boven, 1991*a*). It is mandatory to monitor specific properties, such as volume doubling time and histology, as occasionally tumour tissue may be extensively invaded by mouse stromal tissue or, in the case of one of our STS xenografts, completely replaced by mouse lymphoproliferative cells (Boven, 1991*a*).

Nine STS xenografts were established from patients that needed chemotherapy in the course of their disease. These xenografts indicated that doxorubicin, cyclophosphamide, ifosfamide and vincristine had low activity in this tumour type, whereas etoposide was inactive. In general, the results reflect clinical findings, in which the response rates for single agents are for doxorubicin 13–34%, cyclophosphamide 0–16%, ifosfamide 7–38%, vincristine 0–40% and etoposide 6–16% (Demetri and Elias, 1995). Budach et al (1994) have tested a single dose of doxorubicin (10 mg $kg^{-1}$ i.v.) in 16 STS xenografts and the sensitivity (calculated as specific growth delay >3) was only 13%. For a single dose of ifosfamide (350 mg $kg^{-1}$ i.p.), however, the sensitivity was reported to be 63%. It is always difficult to compare drug efficacy data with those obtained by other investigators, when the methodology is not the same. In this respect, a number of European institutes familiar with the nude mouse–human tumour model have defined guidelines for 'preclinical' phase II studies (Boven et al, 1988). Subsequent studies have revealed that screening of new drugs in a disease-oriented approach becomes feasible with the use of these guidelines (Boven et al, 1992; Langdon et al, 1994). Our panel of human STS xenografts appears to be a valuable addition to the system of secondary drug testing.

Human tumour xenografts are an elegant tool to compare the efficacy of new analogues with that of the parent compounds (Boven, 1991*b*). There are, however, some disadvantages related to differences between species, because the pharmacology and the side-effects of the analogue may follow a different pattern than those of the parent compound. Perhaps ifosfamide is such a compound because, unlike in STS patients, we could not confirm its superiority to cyclophosphamide. This may be explained by differences in drug doses tolerated which was for ifosfamide 1.7-fold higher than for cyclophosphamide in the mouse, whereas ifosfamide in patients can be dosed five times higher than cyclophosphamide upon mesna uroprotection. Treatment of STS xenografts with higher doses of ifosfamide may result in a higher response rate as indicated by the 63% sensitivity in 16 xenografts treated with a single dose of 350 mg $kg^{-1}$ i.p. by Budach et al (1994).

Adult STS can be considered as a moderately chemoresponsive tumour type in the clinic. The majority of patients with advanced disease will not respond or will show progressive disease regardless of the type of chemotherapy. Intrinsic resistance against doxorubicin, vincristine or etoposide does not seem to be related to *MDR1* gene expression as analysed in our STS xenografts. For adult STS in the clinic, a clear correlation between *MDR1* and a poor response to chemotherapy has also not been demonstrated (Stein et al, 1996).

Most studies on reduced topoisomerase II mRNA levels, reduced enzyme activity or mutated topoisomerase II have been carried out in human malignant cell lines selected for resistance against epipodophyllotoxins or anthracyclines (Nitiis and Beck, 1996). Fry et al (1991) have shown that unselected cell lines from testicular cancer had a higher capacity to induce topoisomerase II-mediated DNA strand breaks than bladder cancer cell lines. In cell lines from small-cell lung cancer, it has been found that topoisomerase II catalytic activity and topoisomerase II nuclear protein content were higher than in non-small-cell lung cancer cell lines (Kasahara et al, 1992). In eight unselected human lung cancer cell lines, Giaccone et al (1992) have found a high correlation between topoisomerase II gene levels and sensitivity to epipodophyllotoxins and doxorubicin. In contrast, topoisomerase IIα mRNA expression did not correlate with the in vitro chemosensitivity of acute lymphoblastic leukaemia in children (Klumper et al, 1995). We did not find a relationship between topoisomerase IIα mRNA levels and chemosensivity in STS xenografts. In contrast, the mRNA levels measured were consistently lower than the level in the human ovarian cancer xenograft MRI-H-207, which is highly sensitive to doxorubicin and etoposide.

Cellular glutathione levels are a determinant for sensitivity to alkylating agents and significant glutathione S-transferase activity has been found in tumour cells intrinsically resistant to such drugs (Black and Wolf, 1991; Tew, 1994; Hayes and Pulford, 1995). It has been suggested that elevated glutathione S-transferase π isoenzyme levels are a marker for drug resistance (Black and Wolf, 1991). The roles of glutathione peroxidase and reductase in drug resistance are less clear (Black and Wolf, 1991). Most information on glutathione and glutathione-dependent enzymes in unselected cell lines has been obtained by measuring the sensitivity to cisplatin. In eight human small-cell lung cancer cell lines, Sharma et al (1993) have described that of the four parameters measured only glutathione S-transferase activity correlated with the degree of cisplatin resistance. Hida et al (1993) have reported higher glutathione S-transferase π levels in human lung cancer cell lines with low sensitivity to cisplatin. In cell lines from head and neck cancer, Yellin et al (1994) have found an inverse relationship between cisplatin sensitivity and glutathione contents, but not glutathione S-transferase π mRNA expression. We compared glutathione concentrations and glutathione-dependent enzyme activities with the in vivo sensitivity to cyclophosphamide and ifosfamide, but no correlations could be found. However, levels of glutathione and glutathione S-transferase activity in the human ovarian cancer xenograft were low to intermediate when compared with values in STS lines, whereas in MRI-H-207 tumours the alkylating agents could induce complete remissions.

Our panel of human STS xenografts grown as s.c. tumours reflects the clinic in terms of retention of the histology, human LDH isoenzymes and chemosensitivity pattern. The drug resistance characteristics examined in these xenografts do not explain the reasons for the moderate responsiveness of STS to the various anti-cancer agents tested. Topoisomerase IIα mRNA levels were relatively low, which may partly account for intrinsic resistance against e.g. doxorubicin. DNA repair mechanisms may possibly contribute to the moderate responsiveness to alkylating agents. Doxorubicin resistance has been observed in tumour cells that express the major vault protein encoded by LRP (Slovak et al, 1995) or the multidrug resistance-associated protein MRP (Cole et

© Cancer Research Campaign 1998

al. 1994). Our STS xenografts will probably be of value in elucidating other disease-related resistance mechanisms, towards which compounds may be generated that improve the treatment outcome in this malignancy.

### REFERENCES

Black SM and Wolf CR (1991) The role of glutathione-dependent enzymes in drug resistance. *Pharm Ther* **5**: 139–154

Boven E (1991*a*) Characterization and monitoring. In *The Nude Mouse in Oncology Research*. Boven E and Winograd B (eds), pp. 89–101. CRC Press: Boca Raton, FL

Boven E (1991*b*) Analogs of conventional agents. In *The Nude Mouse in Oncology Research*. Boven E and Winograd B (eds), pp. 185–198. CRC Press: Boca Raton, FL

Boven E, Calame JJ, Molthoff CFM and Pinedo HM (1989) Characterization and chemotherapy of human soft tissue sarcoma lines grown in nude mice. *Strahlenther Onkol* **165**: 538–539

Boven E, Schlüper HMM, Erkelens CAM and Pinedo HM (1990) Doxorubicin compared with related compounds in a nude mouse model for human ovarian cancer. *Eur J Cancer Clin Oncol* **26**: 983–986

Boven E, Winograd B, Fodstad Ø, Lobbezoo MW and Pinedo HM (1988) Preclinical phase II studies in human tumor lines: a European multicenter study. *Eur J Cancer Clin Oncol* **24**: 527–573

Boven E, Winograd B, Berger DP, Dumont P, Braakhuis BJM, Fodstad Ø, Langdon S and Fiebig HH (1992) Phase II preclinical drug screening in human tumor xenografts – a first European multicenter collaborative study. *Cancer Res* **52**: 5940–5947

Bramwell VHC, Mouridsen HT, Santoro A, Blackledge G, Somers R, Verweij J, Dombernowsky P, Onsrud M, Thomas D, Sylvester R and Van Oosterom A (1987) Cyclophosphamide versus ifosfamide: final report of a randomized phase II trial in adult soft tissue sarcomas. *Eur J Cancer Clin Oncol* **23**: 311–321

Broxterman HJ, Feller N, Kuiper CM, Boven E, Versantvoort CHM, Teerlink T and Pinedo HM (1995) Correlation between functional and molecular analysis of *MDR1* P-glycoprotein in human solid-tumor xenografts. *Int J Cancer* **61**: 880–886

Budach W, Budach V, Stuschke M, Schmander B, Reipke P and Scheulen ME (1994) Efficacy of ifosfamide, dacarbazine, doxorubicin and cisplatin in human sarcoma xenografts. *Br J Cancer* **70**: 29–34

Carlberg I and Mannervik B (1985) Glutathione reductase. *Methods Enzymol* **113**: 484–490

Chomczynski P and Sacchi N (1987) Single-step method of RNA isolation by acid guanidinium thiocyanate–phenol–chloroform extraction. *Anal Biochem* **162**: 156–159

Cole SPC, Sparks KE, Fraser K, Loe DW, Grant GE, Wilson GM and Deeley RG (1994) Pharmacological characterization of multidrug resistant MRP-transfected human tumor cells. *Cancer Res* **54**: 5902–5910

Demetri GD and Elias AD (1995) Results of single-agent and combination chemotherapy for advanced soft tissue sarcomas. *Hematol Oncol Clin North Am* **9**: 765–785

Dirix LY and Van Oosterom A (1989) The role of ifosfamide in the treatment of sarcomas. *Semin Oncol* **16**(suppl. 3): 39–45

Feit C, Bartal AH, Fass B, Bushkin Y, Cordon Cardo C and Hirshaut Y (1994) Monoclonal antibodies to human sarcoma and connective tissue differentiation antigens. *Cancer Res* **44**: 5752–5756

Fry AM, Chresta CM, Davies SM, Walker MC, Harris AL, Hartley JA, Masters JRW and Hickson ID (1991) Relationship between topoisomerase II level and chemosensitivity in human tumor cell lines. *Cancer Res* **51**: 6592–6595

Giaccone G, Gazdar AF, Beck H, Zunino F and Capranico G (1992) Multidrug sensitivity phenotype of human lung cancer cells associated with topoisomerase II expression. *Cancer Res* **52**: 1666–1674

Habig WH, Pabst MJ and Jakoby WB (1974) Glutathione S-transferases. The first enzymatic step in mercapturic acid formation. *J Biol Chem* **249**: 7130–7139

Hayes JD and Pulford DJ (1995) The glutathione S-transferase supergene family: regulation of GST and the contribution of the isoenzymes to cancer chemoprevention and drug resistance. *Crit Rev Biochem Mol Biol* **30**: 445–600

Hida T, Ariyoshi Y, Kuwabara M, Sugiura T, Takahashi T, Takahashi T, Hosoda K, Niitsu Y and Ueda R (1993) Glutathione S-transferase π levels in a panel of lung cancer cell lines and its relation to chemo-radiosensitivity. *Jpn J Clin Oncol* **23**: 14–19

Jansen WJM, Pinedo HM, Kuiper CM, Lincke C, Bamberger U, Heckel A and Boven E (1994) Biochemical modulation of 'classical' multidrug resistance by BIBW22BS, a potent derivative of dipyridamole. *Ann Oncol* **5**: 733–739

Kasahara K, Fujiwara Y, Sugimoto Y, Nishio K, Tamura T, Matsuda T and Saijo N (1992) Determinants of response to the DNA topoisomerase II inhibitors doxorubicin and etoposide in human lung cancer cell lines. *J Natl Cancer Inst* **84**: 113–118

Klumper E, Giaccone G, Pieters R, Broekema G, Van Ark-Otte J, Van Wering ER, Kaspers GJL and Veerman AJP (1995) Topoisomerase IIα gene expression in childhood acute lymphoblastic leukemia. *Leukemia* **9**: 1653–1660

Langdon SP, Hendriks HR, Braakhuis BJM, Pratesi G, Berger DP, Fodstad Ø, Fiebig HH and Boven E (1994) Preclinical phase II studies in human tumor xenografts: a European multicenter follow-up study. *Ann Oncol* **5**: 415–422

Lawrence RA and Burk RF (1976) Glutathione peroxidase activity in selenium-deficient rat liver. *Biochem Biophys Res Commun* **71**: 952–958

Molthoff CFM, Calame JJ, Pinedo HM and Boven E (1991) Human ovarian cancer xenografts in nude mice: characterization and analysis of antigen expression. *Int J Cancer* **47**: 72–79

Neuschwander-Tetri BA and Roll FJ (1989) Glutathione measurement by high-performance liquid chromatography separation and fluorometric detection of the glutathione-orthophtalaldehyde adduct. *Anal Biochem* **179**: 236–241

Nitiss JL and Beck WT (1996) Antitopoisomerase drug action and resistance. *Eur J Cancer* **32A**: 958–966

Peters WHM, Boon CEW, Roelofs HMJ, Wobbes T, Nagengast FM and Kremers PG (1992) Expression of drug metabolizing enzymes and p-glycoprotein in colorectal carcinoma and normal mucosa. *Gastroenterology* **103**: 448–455

Santoro A, Tursz T, Mouridsen H, Verweij J, Steward W, Somers R, Buesa J, Casali P, Spooner D, Rankin E, Kirkpatrick A, Van Glabbeke M and Van Oosterom A (1995) Doxorubicin versus CYVADIC versus doxorubicin plus ifosfamide in first-line treatment of advanced soft tissue sarcomas: a randomized study of the European Organization for Research and Treatment of Cancer Soft Tissue and Bone Sarcoma Group. *J Clin Oncol* **13**: 1537–1545

Sharma R, Singhal SS, Srivastava SK, Bajpai KK, Frenkel EP and Awasthi S (1993) Glutathione and glutathione linked enzymes in human small cell lung cancer cell lines. *Cancer Lett* **75**: 111–119

Slevin ML, Clark PI, Joel SP, Malik S, Osborne RJ, Gregory WM, Lowe DG, Reznek RH and Wrigley PFM (1989) A randomized trial to evaluate the effect of schedule on the activity of etoposide in small-cell lung cancer. *J Clin Oncol* **7**: 1333–1340

Slovak ML, Pelkey HJ, Cole SPC, Deeley RG, Greenberger L, De Vries EGE, Broxterman HJ, Scheffer GL and Scheper RJ (1995) The *LRP* gene encoding a major vault protein associated with drug resistance maps proximal to *MRP* on chromosome 16: evidence that chromosome breakage plays a key role in *MRP* or evidence that chromosome breakage plays a key role in *MRP* or *LRP* gene amplification. *Cancer Res* **55**: 4214–4219

Stein U, Shoemaker RH and Schlag PM (1996) *MDR1* gene expression: evaluation of its use as a molecular marker for prognosis and chemotherapy of bone and soft tissue sarcomas. *Eur J Cancer* **32A**: 86–92

Tew KD (1994) Glutathione-associated enzymes in anticancer drug resistance. *Cancer Res* **54**: 4313–4320

WHO (1979) *Handbook for Reporting Results of Cancer Treatment*. Publication No. 48. World Health Organization: Geneva

Winograd B, Boven E, Lobbezoo MW and Pinedo HM (1987) Human tumor xenografts in the nude mouse and their value as test models in anticancer drug development. *In Vivo* **1**: 1–14

Yang JC, Rosenberg SA, Glatstein EJ and Antman KH (1993) Sarcomas of soft tissues. In *Cancer, Principles and Practice of Oncology*. DeVita VT, Hellman S and Rosenberg SA (eds), pp. 1436–1488. JB Lippincott Company: Philadelphia, PA

Yellin SA, Davidson BJ, Pinto JT, Sacks PG, Qiao C and Schantz SP (1994) Relationship of glutathione and glutathione-S-transferase to cisplatin sensitivity in human head and neck squamous carcinoma cell lines. *Cancer Lett* **85**: 223–232

© Cancer Research Campaign 1998